# Community‐Informed Recommendations to Developing Inclusive Dance Opportunities: Engaging Community, Dance, and Rehabilitation Experts Using a Hybrid‐Delphi Method

**DOI:** 10.1111/jar.70060

**Published:** 2025-05-05

**Authors:** Jacqueline C. Ladwig, Kathryn M. Sibley, Jacquie Ripat, Cheryl M. Glazebrook

**Affiliations:** ^1^ Faculty of Kinesiology and Recreation Management University of Manitoba Winnipeg Manitoba Canada; ^2^ Department of Community Health Sciences, Max Rady College of Medicine, Rady Faculty of Health Sciences University of Manitoba Winnipeg Manitoba Canada; ^3^ George and Fay Yee Centre for Healthcare Innovation University of Manitoba Winnipeg Manitoba Canada; ^4^ Department of Occupational Therapy, College of Rehabilitation Sciences University of Manitoba Winnipeg Canada

**Keywords:** constraints model of motor development, dance education, Delphi, inclusive strategies, physical activity, social model of disability

## Abstract

**Background:**

Amongst the growing number of examples of inclusive dance programming, community‐informed recommendations for inclusive dance are scarce. Our purpose was to develop recommendations for inclusive dance with dancers with the lived experience of autism, intellectual, or developmental disability, and the professionals who work with them.

**Method:**

A Hybrid‐Delphi method was used to generate and rank recommendations across three expert groups. The constraints model of motor development and the social model of disability framed three questions around: (i) physical environment and culture, (ii) instruction and strategies, and (iii) dance assistants.

**Results:**

The experts (Community; dancers, support persons/carers (*n* = 5), Rehabilitation (*n* = 6) and Dance (*n* = 7) professionals) agreed to prioritise the community perspective, highlighting the need for ongoing education around inclusive instruction, communication, and sensory considerations.

**Conclusions:**

The centring of community perspectives facilitated the development of a comprehensive list of actionable recommendations to guide inclusive dance instruction in a variety of dance spaces.

**Summary**

- We developed recommendations for inclusive dance that consider instruction (how and what) and the environment (physical space and social culture). The recommendations reflect the perspectives of autistic dancers and dancers who live with intellectual or developmental disability.
- Specific examples of inclusive practices included: creating accessible spaces, using flexible and consistent instruction, welcoming individual ways to participate and communicate, and being proactive regarding sensory considerations.
- The recommendations highlight a need for dance teachers to have a basic understanding of, and access to, ongoing education in disability, barriers and facilitators to participation, and methods of communication (e.g., AAC).
- Other specific strategies emphasise the central role that dance teachers and assistants (volunteer or support staff) have in setting inclusive practices, including the importance of open and explicit communication between the dance teacher, assistant, and dancer.

## 1 Introduction

Dance is a form of expression that spans cultural and socioeconomic contexts (Fancourt and Finn 2019). An ableist history of dance has influenced societal perspectives of who is a dancer, what dance looks like, and how it is performed, across generations (Albright 2011; Aujla and Redding 2013; McCarthy‐Brown 2017). Ableism is pervasive, powerful, and elusive, and therefore continues to restrict how persons who live with disability participate in society broadly (Kattai 2023; Taussig 2020). This history continues to inform how dance is taught and the structure of the dance environment (Anderson 2020; McCarthy‐Brown 2017). Within the dance community, ableist assumptions of who and what bodies are defined as a dancer, along with the lack of disabled bodies in dance, contribute to a lack of confidence around how to create inclusive dance spaces and instruction for persons who live with autism, intellectual, or developmental disability (Aujla and Redding 2013; Hansen and Philo 2007; McCarthy‐Brown 2017). Moreover, adapting the content of a dance class and environment requires discipline‐specific knowledge (e.g., dance form) balanced with an understanding of the experiences of dancers who live with disabilities and the ways in which the dancers may require assistance (Whatley 2008). Formal training or licensing requirements are not required to teach dance in North America. In Canada, dance curricula exist in both dance and education contexts (ADAPT 2023; CSC 2019; PHEC 2023; RAD 2023). Accredited teacher training programs within dance institutions, organisations, and dance societies commonly include anatomy and health education; however, the fundamentals of disability and inclusive teaching strategies are often absent from required coursework (ADAPT 2023; CSC 2019; PHEC 2023; RAD 2023). Importantly, these curricula do not include guidelines for inclusive dance instruction and dance environments that are informed by experts.

In the absence of specific curricula and/or guidelines, expert recommendations from the community can help dance educators, schools, and organisations grow their collective understanding of autism, intellectual and developmental disability specific to instruction and inclusion. Experts here are defined as persons within the intellectual and developmental disability community, including individuals with lived experiences, dance educators with experience creating or adapting inclusive programming, and rehabilitation professionals. Thus, the aim of this study was to systematically develop recommendations to foster inclusive dance environments through the lens of experts who have experience with, or identify as living with autism, intellectual, or developmental disability.

Over the past 20 years, research interest in the field of dance and disability has grown. The potential physical and social benefits of dance for adults who identify as living with autism, intellectual, or developmental disability are supported by current literature (Aujla and Needham‐Beck 2020; DiPasquale and Kelberman 2018; Teixeira‐Machado and DeSantana 2019; Terada et al. 2017). However, literature that explores the experiences of adult populations who identify as living with autism, intellectual, or developmental disability in dance is limited (Ladwig et al. 2023). What limited literature there is focuses on understanding the physiological (DiPasquale and Kelberman 2018; Teixeira‐Machado and DeSantana 2019; Terada et al. 2017) and psychosocial benefits (Aujla and Needham‐Beck 2020; Mastrominico et al. 2018) of dance for adults who identify as living with autism, intellectual, or developmental disability. Importantly, the ways dance has been adapted for persons who identify as living with autism, intellectual, or developmental disability and how, or if, their perspectives were included in the dance interventions explored in the literature remain unclear (Ladwig et al. 2023). In the current context of dance education, community‐informed recommendations on how to develop and foster inclusive instruction and dance environments are lacking, particularly those that are based on the perspectives and voices of dancers who identify as living with autism, intellectual, or developmental disability, and those who support them (i.e., support persons or carers).

When considering what strategies and methods are best to include in recommendations, we assert that the lived experience of dancers who identify as living with autism, intellectual, or developmental disability can only be communicated by the dancers themselves, that is, the knowers (Oliver and Barnes 2010; Stone and Priestley 1996). In our recent scoping review, we reported that although persons with a range of functional levels and varied communication methods have been included as participants in studies using dance interventions, descriptions of how dance interventions were adapted to include those with more complex communication and functional capabilities were lacking (Ladwig et al. 2023). Further, the viewpoints and perspectives of support persons/carers were often not sought (Ladwig et al. 2023).

The development of the recommendations should recognise and respect both the viewpoint and autonomy of persons with autism, intellectual, or developmental disability, and recognise the voice of support persons and carers as separate (Leadbitter et al. 2021). Support persons/carers who participate in dance alongside the dancers could provide insight into the level of training they may require, the ways physical assistance may be most effective (e.g., ways to engage dancers who are quadriplegic and how best to encourage independent movement), and options for communication strategies. In the present study, the perspectives of both parties (dancers and support persons/carers) were included as their combined perspectives may provide important insights that could drive how verbal and physical instructions are provided. The specific study objectives were to: (i) collect and articulate expert (community, dance, and rehabilitation) perspectives on methods of instruction and strategies for adapting dance and dance environments, and (ii) formulate the methods and strategies into a set of prioritised recommendations to be shared with the dance community broadly. We planned for the recommendations to be offered as a guide for the development of inclusive pedagogical approaches to dance and movement education environments and interventions.

## 2 Method

We used a Hybrid‐Delphi method to gather and prioritise expert perspectives (Landeta et al. 2011). The Hybrid‐Delphi method is a systematic and iterative method of assembling viewpoints of experts on a specific subject using a combination of focus groups that engage a modified nominal group technique (Landeta et al. 2011) and classical Delphi techniques to rank the statements. To facilitate the process of developing the expert responses during the focus groups, a modified nominal group technique was used where: (i) we shared three questions with the participants in advance of the focus group so that they could consider the questions and prepare their initial responses offline, (ii) we asked participants to share their initial or prepared responses during the focus group, and (iii) we facilitated discussion and revision of the prepared responses (Landeta et al. 2011). In this way, the Hybrid‐Delphi technique promotes participant engagement and addresses the lack of opportunity for group interaction that occurs in the classical Delphi in which the procedure typically occurs solely by email (Hasson et al. 2000; Mead and Moseley 2000). All procedures were approved by the Research Ethics Board 1 of the University of Manitoba, in accordance with the Declaration of Helsinki (2002).

## 3 The Framework

To frame the questions and contextualise expert recommendations we used the social model of disability and the constraints model of motor development (Newell 1986; Oliver 2013). The constraints model proposes that our environment, instruction, as well as our personal physical and emotional characteristics shape our motor development (Newell 1986), whereas the social model considers the ways that societal structures and policies disable the individual (Oliver 2013). Together the models address the reciprocal relationship between the physical and emotional aspects of the individual, the physical task and instructions, and the environment's social, cultural, and physical elements (Newell 1986; Oliver 2013). Integrating these models centres the person and their lived experience to consider what to do, rather than enlisting a healthcare model where the focus is often on ‘fixing’ or ‘correcting’ impairments. For the current study the models were used to consider the relationship between physical, perceptual, social, and affective constructs within the dance class and dance culture that may limit the participation of the dancers. Specifically, the models were used in two ways: to develop the focus group questions (see Table 1) and to contextualise the expert recommendations. With the goal of sharing informed and actionable recommendations, these combined models centred the characteristics, capabilities, and experiences of the dancers and addressed in what ways and how the instructions and environment (culture and physical) may be adapted to accept and embrace the physical, psychological, and social needs of the dancers (Getchell and Gagen 2006; Leadbitter et al. 2021; Oliver and Barnes 2012).

**TABLE 1:** The three questions.

1.	What considerations do you believe should be prioritised regarding the class structure and the environment of the adapted/inclusive dance class (e.g., methods of communication, setup of the space, use of assistants, use of music, flow and/or pace of the class, etc.)?
2.	What teaching strategies (e.g., verbal and/or physical cues, how steps are modified, how modifications are presented, groupings and groups performing for one another, etc.) do you believe should be included?
3.	What forms of information, assistive strategies, and/or training do you feel should be provided to prepare and support the caregiver or support person for their role?

*Note:* All three questions were posed to each group of experts.

### 3.1 Participants

A purposive approach was used to identify participants from the disability, dance education, and rehabilitation communities. Study information was shared with 15 Canadian disability, arts, and rehabilitation organisations and with 13 experts known to the primary researcher. We defined ‘experts’ as individuals ≥ 18 years of age who identified with one or more of three groups: (1) persons who identify as living with autism, intellectual, or developmental disability and currently participate in dance, (2) persons who identify as support person/carer to an individual who identifies as living with autism, intellectual, or developmental disability in dance, or (3) a dance or rehabilitation professional who has experience working with individuals with autism, intellectual, or developmental disability (see Table 2 for inclusion criteria). In the context of this study, the term ‘assistant’ refers to any individual who has assisted a dancer in class, including support persons/carers hired by the dancer or their family, employed by the dance school, or has volunteered to provide support in a class.

**TABLE 2:** Group inclusion criteria.

Group	Criteria
Community experts	Adult dancers who self‐identified as living with autism, intellectual, or developmental disability and have participated in an adapted/inclusive dance program in the past 6 years. Support person/carers who had been an active participant alongside the individual they support in dance or have supported an individual through presence and observation alone.
Dance experts	Dance educators who, in the previous 6 years, have taught adults who identify as living with autism, intellectual, or developmental disability in either inclusive or traditional dance settings, such as academic (high school or post‐secondary), dance studios, adult day programs, or in the community. No specific education or training experience was required.
Rehabilitation experts	Rehabilitation professionals, including physiotherapists and occupational therapists, who have currently work with or in the past 6 years have worked with individuals living with autism, intellectual, or developmental disability.

## 4 Data Collection and Analysis

**FIGURE 1:**
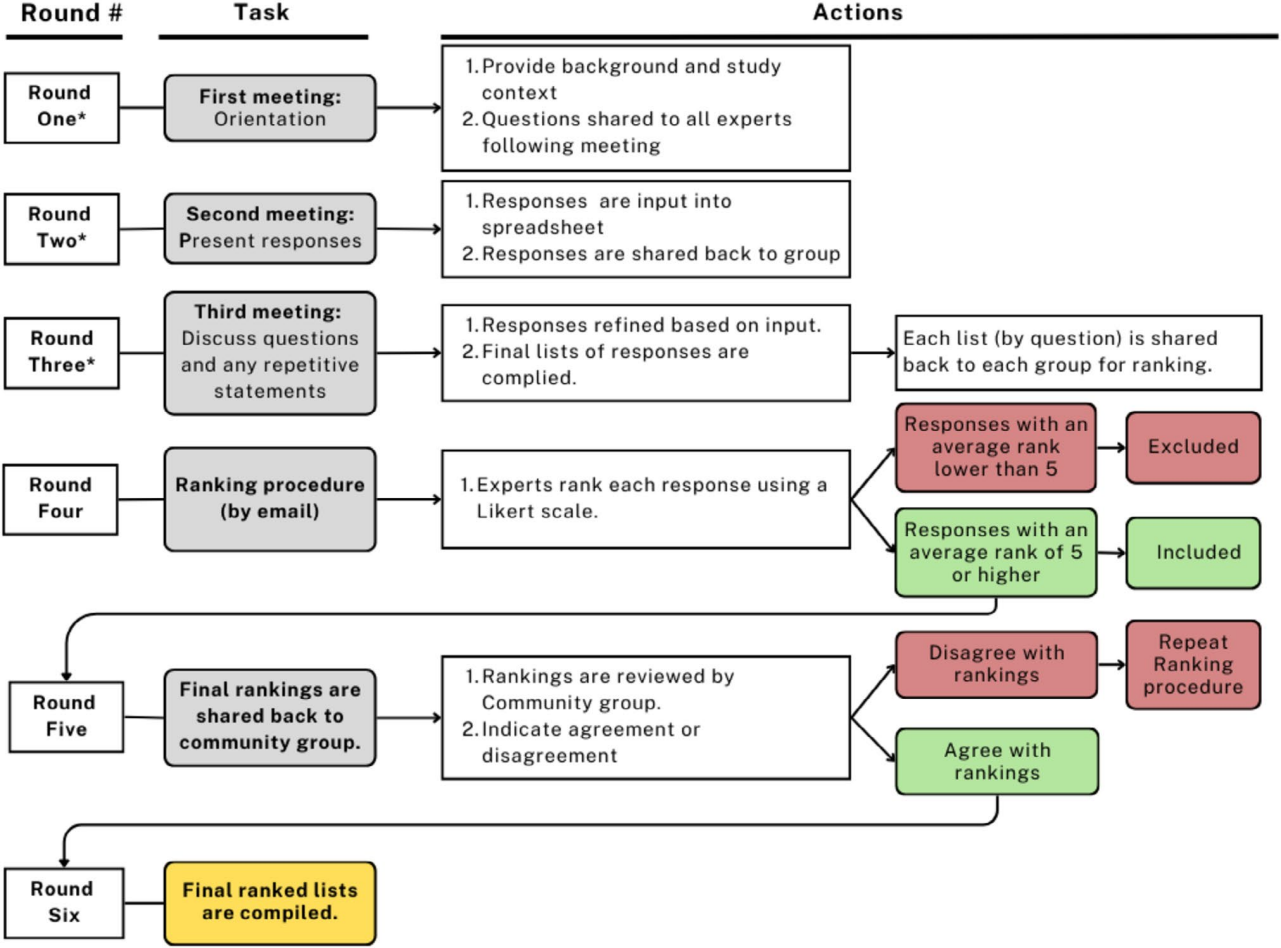
Overview of the procedure. *Note:* (*) rounds 1, 2, and 3 occurred virtually.

An overview of the procedure is provided in Figure 1. Three expert groups were determined based on the participants' lived experience and/or training. We chose to hold separate focus groups (homogenous) with each group. This decision was made with the intent to address potential power differentials, foster group cohesion, limit potential ‘Zoom fatigue’, and to facilitate scheduling. All focus groups were held virtually on the Zoom platform and approximately 75 min in length. All e‐surveys were created using Microsoft Forms. A research assistant attended all focus groups and was tasked with taking notes, monitoring questions, and ensuring that each participant had the opportunity to share their responses and perspectives. Following each focus group, the researcher and assistant met to debrief any concerns or ideas that emerged during the session.

### 4.1 Round One

In round one, the first meeting was conducted with each group (see Figure 1). To orient the participants to the study, the first author provided context for the research and summarised the process and theoretical approach. These first steps were done with the goal of preparing the participants for the focus groups and enhancing participant response rates (Humphrey‐Murto and de Wit 2019; Mead and Moseley 2000). To encourage group connection and foster the commitment of each group, the participants were asked during this first meeting to share their thoughts, doubts, and ideas, and to reflect on the study purpose and procedure (Landeta et al. 2011). To prepare for the next round and encourage participant reflection, each group received the questions (see Table 1) following the first meeting (within 24 h) and were encouraged to write out their responses before round two.

### 4.2 Round Two

In round two, a virtual focus group was conducted with each expert group. The researcher began by reviewing the process of the focus group, presented each question to the group, and each participant presented their responses to each question (Landeta et al. 2011). We used a round table technique to ensure each participant had an opportunity to contribute and share their most important responses to each question. Participants were also directed that they would have the opportunity and time to revise their responses during the focus group. Following this focus group, participants were asked to share their complete list of responses with the research assistant by email.

#### 4.2.1 List Preparation and Analysis

**FIGURE 2:**
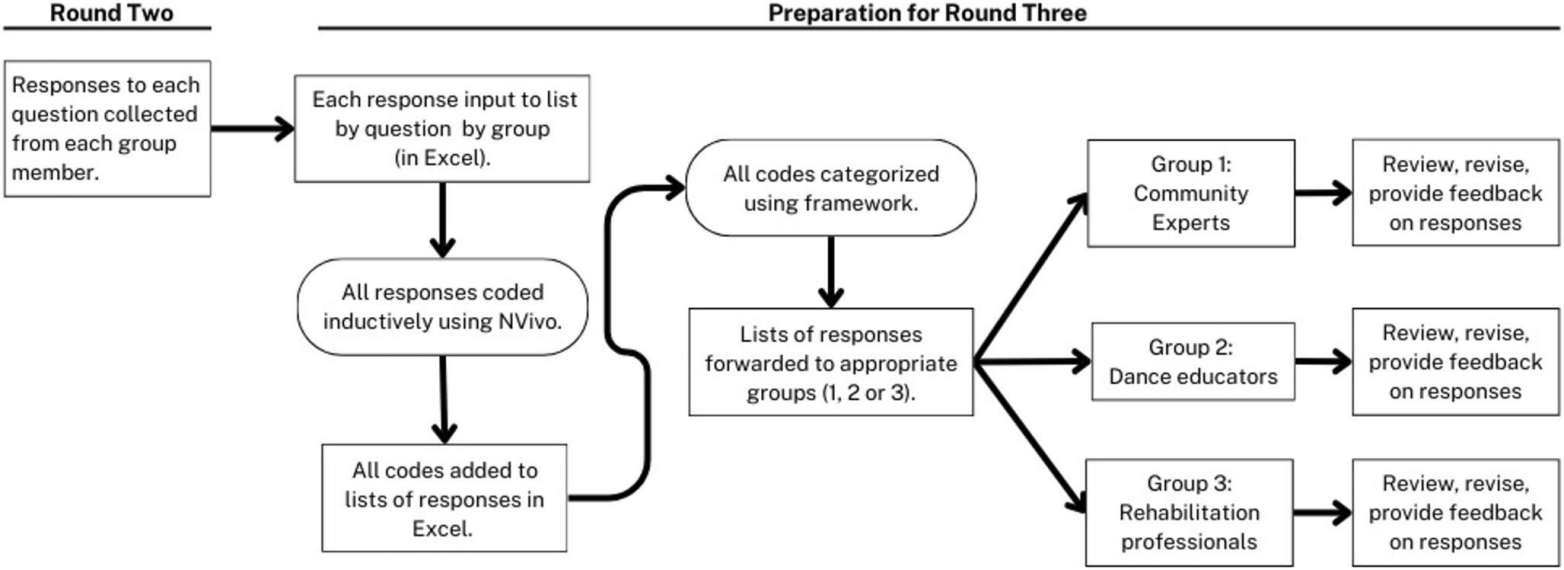
Analysis procedure (rounds two and three).

Prior to round three, each list of responses to each question, and from each group, was aggregated, de‐identified, and input into separate Excel spreadsheets; each spreadsheet was then uploaded into NVivo (see Figure 2). To capture the main focus of each response, we used inductive coding using NVivo. The codes were then categorised using concepts from the model of constraints and the social model of disability (e.g., categories such as instruction, environment, inclusion, and adaptation). All codes were added to each group's lists of responses by question in Excel and the spreadsheet for each question was then sorted by code. To enhance credibility, accuracy and panellist engagement, each group's responses were shared back to each participant for their review, revision, and feedback using an individual Excel shared file links. Participants were given 2 weeks to complete this task.

### 4.3 Round Three

In round three, the lists of responses generated to this point were reviewed with each group in a virtual focus group setting. The researcher presented questions that arose through the feedback and review process (in round two) for discussion. Participants suggested responses they felt could be collapsed. From this feedback, the researcher further collapsed the responses. This process was completed using Excel spreadsheets to track the original statements and facilitate sorting by codes.

### 4.4 Round Four

In round four the reduced statements were shared back to the corresponding group participants for ranking by e‐survey using Forms (see Figure 1). Participants were asked to prioritise each statement using a seven‐point Likert scale, where seven was the highest priority and one was the lowest. Upon completion of the ranking procedure, all mean scores were calculated in an Excel spreadsheet and the final lists for each group were ordered accordingly. The ranked results and mean scores for each statement were shared back to participants using individual Excel shared file links. Participants were asked to indicate their agreement or disagreement with each ranking and, should they disagree with a ranking, asked to provide additional feedback. Participants were given a period of 2 weeks to respond to each e‐survey. To encourage a response rate of ≥ 70%, panellists received two email or text message reminders before the deadline (Hasson et al. 2000).

### 4.5 Round Five

In round five, the researcher reviewed the rankings with each community group participant individually, either in‐person or virtually (see Figure 1). Each participant was asked to indicate their agreement or disagreement with the rankings and was asked for their feedback on the statements. The ranking process was repeated if consensus was not reached at this stage. If agreement was reached, then the mean scores for each statement guided the compilation of the final list of recommendations.

Consensus was defined as a median score of 71% or higher (≥ a rank of 5), 80% agreement of the rankings from the community expert group, or the completion of no more than the described two rounds of statement rankings, whichever was attained first. Consensus was required for inclusion in the final list of recommendations.

### 4.6 Round Six

The final lists of statements from each group were integrated into one list per question, forming three lists (i.e., recommendations for (i) adapting the class structure and environment, (ii) inclusive instruction, and (iii) information for assistants). Statements with mean scores of less than five were removed and saved to a separate list in case the panellists wished to return to a statement (Mead and Moseley 2000). The final lists were shared back to all participants with an invitation to provide feedback (e.g., on clarity, formatting).

## 5 Results

### 5.1 Participants and Attrition

Twenty‐one Canadian experts consented to participate in the study: community experts (*n* = 5), dance experts (*n* = 9), and rehabilitation experts (*n* = 7). See Table 3 for group characteristics. Participation and response rates were > 80% across all rounds of the Delphi procedure. Attrition primarily occurred due to personal schedules (*n* = 3) and those lost to follow up (*n* = 1). Of the experts (across the three groups) who remained in the sample and were engaged through study completion (*n* = 18), 94% self‐identified as female, 83% self‐identified as white, 61% resided in Manitoba, while the remainder resided in Ontario, Alberta, and British Columbia, 18% self‐identified as autistic, and 82% had completed an undergraduate, master's, or doctoral degree.

**TABLE 3:** Group characteristics.

Characteristic	Description
Community group (*n* = 6)	Identified as dancers (*n* = 3); support a dancer with a NDD (*n* = 3)
Age range	25–64 years
Dance experience	None (*n* = 2); more than 10 years (*n* = 1)
Support experience	1–3 years (*n* = 2) to 4–5 years (*n* = 1)
Location	Manitoba and British Columbia
Dance educators (*n* = 9)	Taught in a studio setting (*n* = 6); in community settings; (*n* = 3)
Age range	25–64 years
Experience in relation to disability	1–3 years (*n* = 2) to 10 or more years (*n* = 7)
Location	Manitoba, Ontario, and Alberta
Rehabilitation professionals (*n* = 8)	Occupational or physical therapists in clinical settings (*n* = 6); community or school setting (*n* = 2)
Age range	25–64 years
Experience	1–3 years (*n* = 3) to 10 or more years (*n* = 4)
Dance experience	None (*n* = 2); recreational experience (*n* = 6); with teaching certifications (*n* = 2)
Location	Manitoba, Ontario, British Columbia

### 5.2 Generation of Responses

**FIGURE 3:**
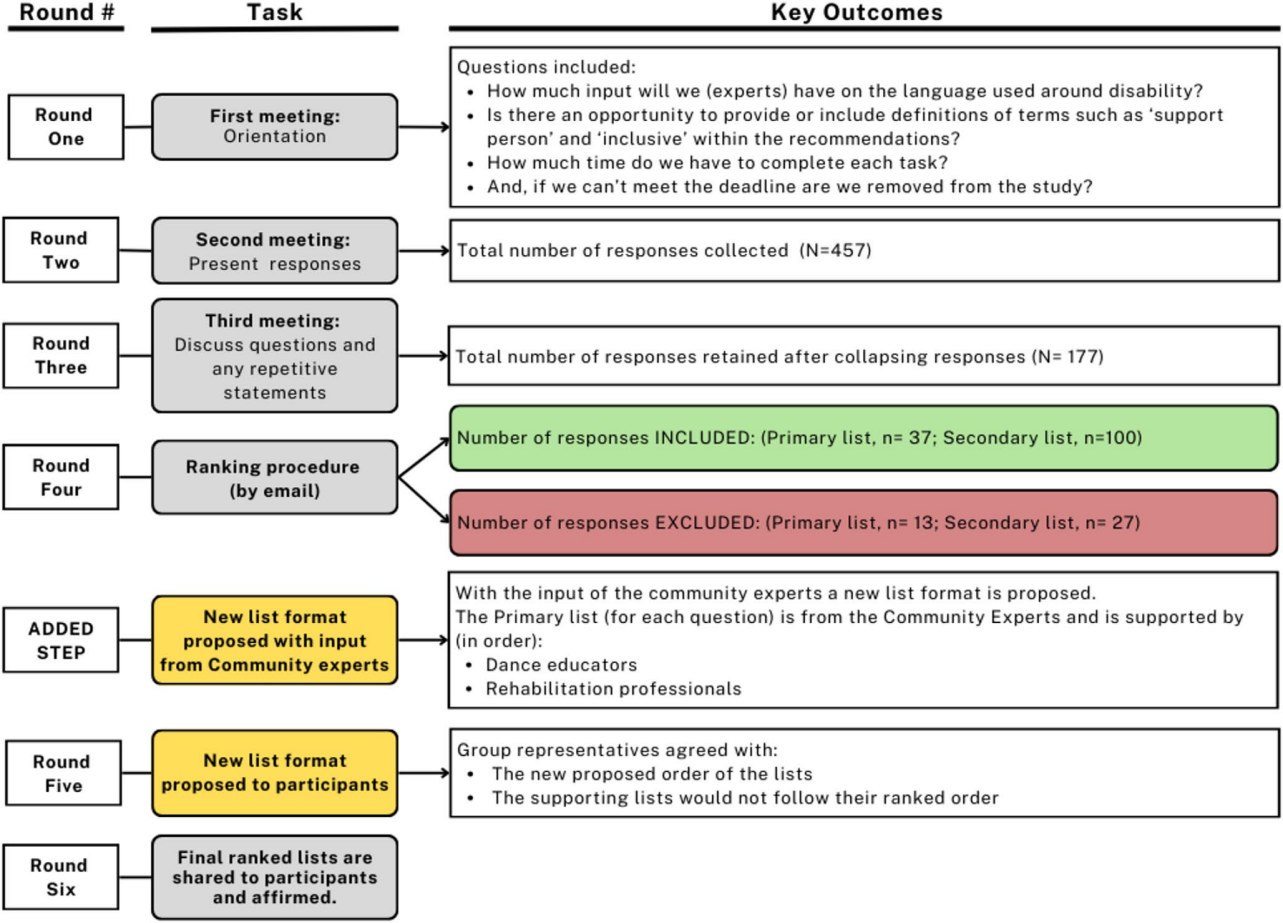
Key outcomes of each round.

In round one, each group participated in the initial virtual meeting for approximately 75 min. Questions that arose are included in Figure 3. Reflecting on these first meetings, each group was supportive of the study purpose, the inclusion of the perspectives of the three expert groups and was particularly supportive of the a priori plan to centre the community expert voices. All subsequent meetings were approximately 90 min in duration.

In round two, participants identified and shared what they felt were their most important responses and provided relevant contextual information (*N* = 457 responses; see Figure 3). Participants suggested using some of the responses as a preamble to the lists whereas others noted that the ‘fun’ factor had not been addressed and suggested the inclusion of a related statement in a preamble. Following round two, each participant reviewed the responses generated for each question within their group and provided feedback regarding language (90% response rate). No changes in wording or language were required.

At round three, the individual lists of group responses were reduced by as much as 58%. Due to the number of responses, the rehabilitation group was unable to complete the task for all three lists in one meeting. At the groups' suggestion, the researcher reviewed the remaining lists. To ensure transparency and clarity of the participants' intended messages, the collapsed statements were shared back to the rehabilitation group alongside the original statements to ensure that they could clearly identify any changes made to the statements (an example of this process can be found in the Supporting Information). Feedback received from all groups included suggestions around language and specific statements to be collapsed. After the feedback was incorporated, the revised statements were shared back to those participants who had provided feedback to ensure that their input was captured and that the intended message of the original statements was maintained in the revised statements (see Figure 3).

In round four, there was an 85% response rate for the ranking by e‐survey. There were no disagreements with the final rankings across the expert groups, so an additional round of ranking was not required (see Figure 3).

Following round four, the primary author identified that there were common categories across the groups that resulted in overlapping statements between groups. The language differed between the lists as the rehabilitation and dance experts used more technical, action‐oriented language with explicit details specific to their professions. A review of the lists of recommendations from each group highlighted a shared focus for each question across the groups. For example, in question one (environment and class structure) the shared focus across the groups included the space, class size, accessibility, flexibility of instruction, sensory needs, fostering participation, observation, and communication. The level of explicit detail provided by the dance and rehabilitation experts highlighted and supported the community expert responses by providing explicit details and examples. To ensure that the community expert contributions were not overshadowed by the level of detail provided by the rehabilitation and dance experts, it was proposed to each group that the community expert lists serve as the primary recommendations (see the ‘Added step’ in Figure 3). The responses from the rehabilitation and dance experts were included in a separate supporting list. As this proposition deviated from what was described to participants at the outset of the study, it was agreed that the community experts would be consulted first, and, if they agreed, the remaining expert groups would then be consulted on this change.

At round five, all community experts agreed with the proposition to maintain their responses as the primary list. The community experts also suggested that the supporting lists of responses be prioritised in the following order: (1) dance experts followed by (2) rehabilitation professionals. As this proposed change deviated from what was previously described to participants, this change was confirmed with available participants from each group (rehabilitation, *n* = 2; dance, *n* = 2) by phone. It was explicitly noted that shifting their responses to a supporting list meant that their responses would not be listed in ranked order (see Figure 3). All participants agreed to the supporting list, noting that this change shifts away from a biomedical focus to a person‐centred focus.

The final lists were shared back to the participants in round six (see Figure 3). Participants could choose to provide additional feedback. Three participants affirmed the final lists, noting that the homogeneity of the community group as a possible limitation and a concern that the lists might make it seem ‘simple’ to create and teach inclusive programming. The latter concern was resolved through a discussion around how the presentation of the three lists together highlights the variables to be considered when working toward inclusion, as opposed to oversimplifying the process of inclusion. Summaries of the primary recommendations are provided in Tables 4, 5, 6. The lists of supporting recommendations are found in Tables [Link], [Link], [Link].

**TABLE 4:** Primary ranked recommendations: question 1.

(Q1) primary recommendations for adapting the class structure and environment
Understand and anticipate that changing the location of the class and sounds within and surrounding the class can be a source of anxiety and distraction. See Table S7: (1) The space^a^
2 Smaller class sizes (< 10 persons) and organised by age (children and adults separate) are immediately most inclusive. See Table S7: (2) Class sizes^a^
3 Ensure that both the building, dance space (studio), bathrooms, and changing areas are accessible. See Table S7: (3) Accessibility^a^
4 To reduce uncertainty and anxiety for the dancers follow a consistent class structure, remain flexible, and respond to the needs of the group. See Table S7: (4) Consistency and flexibility of instruction^a^
5 Design instruction to deliberately incorporate adaptive sensory transitions such as adjusting the volume of music or dimming the lighting to help direct and maintain dancers' attention. See Table S7: (5) Consider sensory needs and transitions^a^
6 Encourage all dancers to actively participate in their own way. See Table S7: (6) Facilitate participation^a^
7 To reduce anxiety around being observed provide options to cover viewing windows and mirrors and provide a waiting area outside of the studio. See Table S7: (7) Observation^a^
8 Clearly communicate class structure, expectations, and changes before the class to both dancers and families/support persons/carers. See Table S7: (8) Communication^a^

*Note:* N/S indicates that the recommendation is not linked to a specific supporting recommendation.

^a^ References the related supporting recommendations provided by dance educator and rehabilitation groups.

**TABLE 5:** Primary ranked recommendations: question 2.

(Q2) primary recommendations for inclusive instruction
Provide and demonstrate adaptations for the different abilities and capabilities of all the dancers, or if a dancer is struggling. See Table S8: (1) Adaptation for different abilities of dancers^a^
2 Acknowledge progress and effort through honest feedback and direction. See Table S8: (2) Feedback and direction^a^
3 Teachers should have a background in dance and are able/willing to move with the dancers during the class (active), rather than just sit and observe (passive). See Table S8: (3) Active vs. Passive instruction^a^
4 Lead with empathy and understanding. Be open to adjusting the class plan and/or structure when needed. See Table S8: (4) Flexible instruction^a^
5 It is critical for teachers and assistants to gain an understanding of the general characteristics of the dancers' diagnoses and the experience of disability. See Table S8: (5) Understanding disability^a^
6 Allow dancers to choose to rest when needed. See Table S8: (6) Attend to dancers physical & social cues^a^
7 The incorporation of breathing exercises on a regular basis can help to regulate both anxiety and heart rate. See Table S8: (7) Incorporate regulation skills^a^
8 When offering help do so in an unobtrusive way to ensure that no dancer feels left out or singled out. See Table S8: (8) How to assist dancers in class^a^
9 Ensure that the pace of the class progresses in a timely manner (i.e., do not be swayed by students talking during instruction). See Table S8: (9) Pace of the class^a^
10 It is important to understand that the dancers experience good and bad days; sometimes there are no solutions, just let all feelings be valid. See Table S8: (10) Understanding & self‐regulation^a^
11 It is helpful when teachers and assistants are positive and energetic. Make the class fun, yet balanced with purposeful activities and provide respectful, direct guidance. See Table S8: (11) Positive and energetic^a^
12 Engage strategies that encourage participation and support learning. See Table S8: (12) Support participation & learning^a^
13 For some individuals it can be helpful to talk them through what is happening to their body if injured or experiencing dysregulation, and soforth. See Table S8: (13) Be prepared^a^
14 The teacher needs to be aware that their tone of voice or how they look at a dancer can be encouraging or discouraging. See Table S8: (14) Be self‐aware^a^
15 Cultivate a culture of acceptance that allows and welcomes the dancers' voices, opinions, and perspectives on issues they feel are important in class. See Table S8: (15) Cultivate a culture of acceptance^a^
16 Integrate the principle of inclusivity into class instruction so that dancers understand that they are also building life skills such as self‐advocacy, resilience, and adaptability. N/A
17 To foster communication and flow of the class incorporate visual cues (signs & schedules) and hand signals (e.g., follow along, please be quiet, listen). See Table S8: (17) Visibility & visual communication^a^
18 Some behaviour related to autism, intellectual, or developmental disability may cause anxiety or intimidate other dancers. To foster familiarity and connection incorporate ways for students to get to know one another during the class. See Table S8: (18) Foster familiarity and understanding^a^

*Note:* N/S indicates that the recommendation is not linked to a specific supporting recommendation.

^a^ References the related supporting recommendations provided by dance educators and rehabilitation groups.

**TABLE 6:** Primary ranked recommendations: question 3.

(Q3) primary recommended information for assistants^b^
It is important that assistants^b^ are an active participant in the class, rather than being a passive observer. See Table S9: (1) Be an active assistant^a^
2 It is critical that support persons/carers and assistants^b^ allow dancers to initiate movement before jumping in to assist with movements. See Table S9: (2) Dance with, not for^a^
3 Provide and encourage opportunities for assistants^b^ and support persons/carers to share strategies and ways to support dancers. See Table S9: (3) Provide support and ongoing learning^a^
4 It is most helpful when assistants model the teacher, the dance/activities, a sense of humour and positive attitude. See Table S9: (4) Model behaviour^a^
5 Assistants^b^ must understand that every other participant in a classroom deserves to have their time and effort considered and respected; it is not acceptable to presume that disrupting an entire class is ‘no big deal’ because ‘it's just one question’. See Table S9: (5) Be respectful^a^
6 Provide regular learning opportunities for all leaders within the dance community (teachers, assistants, directors, dancers, parents, etc.). See Table S9: (6) Ongoing learning^a^
7 Ask in advance how each dancer prefers to communicate best (any specific cues that should be used, ASL, etc.). See Table S9: (7) Communication^a^
8 An agreement to abide by protocols and expectations (such as anti‐bullying policies) ought to be a prerequisite for participation. It is the responsibility of teachers to communicate and uphold this structure. N/A

*Note:* N/A indicates that there are no supporting recommendations for this recommendation. N/S indicates that the recommendation is not linked to a specific supporting recommendation.

^a^ References the related supporting recommendations provided by dance educator and rehabilitation groups.

^b^ The term assistant is defined as persons who are hired as teaching assistants or volunteer as a class assistant. Support persons are defined as carers (employed by a day program, individual, or family) or family participants attending with the dancer.

## 6 Discussion

The primary objective of this study was to develop a community‐informed list of recommendations for inclusive dance that was informed directly by community experts (dancers who identify as living with autism, intellectual, or developmental disability, and those who support them), dance educators, and rehabilitation professionals. We intentionally centred the experiences and perspectives of the community experts and used the constraints model of motor development and social model of disability to consider how and what aspects of dance instruction, environment and culture can be adapted or modified to anticipate known needs of the dancer (Newell 1986; Oliver 2013). Through the insights of the 18 experts, we systematically developed 34 primary recommendations (see Tables 4, 5, 6) and 111 supporting recommendations that consist of specific actions that can be used to guide inclusive instruction and support inclusive dance environments (see Tables [Link], [Link], [Link]). The ranked responses of each group were synthesised into two sets of lists: (i) community expert recommendations, and (ii) supporting recommendations as proposed by the dance and rehabilitation professionals (see Tables [Link], [Link], [Link]).

### 6.1 The Class Structure and Environment

The community experts' recommendations highlighted the elements critical to their experience, including access, flexibility, consistency, participation, communication, and being proactive regarding sensory considerations (see Table 4). Likewise, the dance and rehabilitation experts' recommendations included explicit actions to address sensory processing (light, sound, proprioceptive) in relation to the space and instruction, the functional accessibility of the space and building, ways to promote participation through instruction, and engaging a structured yet flexible class structure, and communication (see Table S7). The applied nature of these recommendations lies in the supporting list of recommendations derived from the practical experiences of the dance and rehabilitation professionals (see Table S7). Previous literature has proposed barriers to participation including lack of building access, as well as spaces and instruction that account for students' sensory needs (Aujla and Redding 2013; Suppo and Swank 2019). The supporting list builds on the previous literature and directly responds to known barriers to participation such as accessibility of buildings and dance spaces by incorporating concrete actions and contextual information around instruction and the studio environment (Whatley 2007). In summary, central to the design of inclusive dance opportunities is a foundational understanding of disability, the ways in which instruction is designed, and if and how expectations of movement performance can be adjusted.

### 6.2 Inclusive Instruction

Each expert group was asked to consider instruction and what strategies might need to be included/adapted and how to include those strategies in a dance experience. Congruent with existing literature (Aujla and Redding 2013; Suppo and Swank 2019; Whatley 2007), an underlying theme across the recommendations was the need for the provision of, and a base competence with, foundational knowledge of disability, including diagnoses and behaviours, barriers and facilitators to participation, methods of communication, and the importance of the teachers' role in creating an inclusive dance experience more broadly (see Table 5 and Table S8). The experts also prioritised recommendations around characteristics of the teachers and assistants, such as being encouraging, providing clear and direct instruction, being honest in their feedback, demonstrating a positive and energetic attitude, actively dancing with the dancers rather than passively observing, and communicating clearly with the dancers (see Table 5). These findings support the suggestions of Morris et al. (2015) and Suppo and Swank (2019) regarding important characteristics of instruction and assistants. For example, providing explicit training opportunities for assistants so that they can become familiar with common characteristics of the dancers, and the importance of being enthusiastic. Additionally, existing literature supports using a discovery‐oriented, collaborative shared‐learning approach for both teachers and assistants (Morris et al. 2015; Suppo and Swank 2019). Taken together, the suggestions around instruction and assistants further highlights the importance of the teacher and assistants' positive energy and open communication with dancers and assistants in inclusive dance settings.

The supporting list of recommendations related to instruction provides the groundwork for developing more inclusive dance instruction and reinforces the importance of teachers and assistants engaging in ongoing skill development specific to disability and accommodations (see Tables S8 and S9). Flexibility of the class structure, including the teacher's approach to instruction generally, was one important instruction strategy. Supported by existing literature, this finding highlights the importance of balancing instructions around technical dance elements with an exploration of dance concepts. This balanced approach of technique and exploration will help ensure that dancers develop a foundational technique within their own physical capabilities and explore the concepts of dance and movement more broadly (Tomasic 2014; Morris et al. 2015; Suppo and Swank 2019).

Another focus of the strategies for class instruction is recognising that progress may look different for dancers who identify as living with autism, intellectual, or developmental disability. For example, it may take more time for a dancer to master a step or movement, or they may perform a movement in an unexpected manner, or they may express their feelings or needs differently (e.g., physical cues), as compared to a dancer who does not have a disability (Aujla and Redding 2013). Understanding, questioning, and reflecting on how a dancer might participate in the dance class is critical to the process of creating inclusive dance spaces. Taking these steps may foster the teacher's ability to proactively guide dancers through their class. For example, in a recent study we asked dancers who identify as living autism, intellectual, or developmental disability about their experiences with, and perspectives on, dance instruction (Ladwig et al. 2024). The dancers expressed that when instruction prioritised dancers' needs, their experiences in dance led to a sense of belonging and purpose. In contrast, the dancers also expressed that when their differences were pointed out by the teacher in front of others, they experienced feelings of isolation, feeling ‘not good enough,’ and lonely. This finding demonstrates how a teacher's communication with dancers can directly impact their sense of belonging and inclusion. Thus, highlighting the importance of the teacher's role in the process of inclusion as the approach they choose can create a culture of acceptance, where instruction begins with the presumption that all individuals/dancers are competent, or conversely can create a culture that excludes and isolates persons who identify as living with autism, intellectual, or developmental disability through their words and actions. The supporting lists of recommendations provided are actionable items (see Table S8) around instruction that demonstrate how instruction can be designed and/or delivered to consider the capabilities of dancers who identify as living with autism, intellectual, or developmental disability. Persons who assist the dancers and/or the teacher directly also play an important role in the dance class. How teachers acknowledge and communicate with the assistants, support staff and/or carers may also influence dancers' experience in, and the culture of, the dance class.

### 6.3 Information for Dance Assistants

As other authors have suggested, it is ideal if the teacher, dance school, or organisation can provide regular volunteer or employed assistants in the class (Morris et al. 2015; Suppo and Swank 2019). For some individuals, it may be helpful to have a known assistant/support person assist them during the class. The supporting recommendations highlight the need for assistant training in advance of or during their participation in a program (Suppo and Swank 2019; see Table S9). These findings outline specific strategies that emphasise the importance of the role of assistants and their ability to impact the dancers' experience, the importance of communication between the teacher, assistant, and dancer, and support ongoing education for all assistants and teachers (see Tables 6 and S9). These insights may also inform how to best incorporate, direct, and train support persons/carers for adapted and/or inclusive dance experiences.

To the best of our knowledge, this is the first study to use a Hybrid‐Delphi method to establish and prioritise expert recommendations on what and how aspects of the dance culture, environment, instruction, and assistance should be considered and/or adapted when designing inclusive dance programming. A strength of this study is the shared recommendations across the three expert groups with the prioritisation of the community experts' perspectives. We explicitly engaged and centred the recommendations on the perspectives of the dancers and those who support them through (i) a priori plan for rankings to be reviewed and agreed upon by the community experts, and (ii) a post hoc revision to present the community expert recommendations as the primary recommendations.

The findings of this study align with existing literature and reflect the need to include the perspectives of the dancers who identify as living with autism, intellectual, or developmental disability, as well as known barriers and facilitators to participation in physical activity for persons who identify as living with autism, intellectual, or developmental disability broadly (Aujla and Redding 2013; Ladwig et al. 2023; Suppo and Swank 2019; Wright et al. 2019). Another strength of this study lies in the applicability of both lists of recommendations across dance and physical activity settings. Importantly, these lists are not intended to be prescriptive in nature; instead, these recommendations are tools to guide educators in a range of movement‐based settings, including, but not limited to, dance schools and studios, community programs, and physical education.

Finally, this study adds to previous reports around a lack of teacher training in relation to disability and movement, specifically the integration of dancers who live with disabilities into physical activity and dance environments (Aujla and Redding 2013; Whatley 2007; Wright et al. 2019). This study provides actions to shift the narrative in dance and physical activity toward normalising difference and inclusive approaches to (dance) instruction (Hansen and Philo 2007). These comprehensive findings may also be used to promote discourse around common practices engaged in dance and dance spaces, and the assumptions made about persons who live with disability in the context of instruction and the culture of the dance community (Aujla and Redding 2013; Leadbitter et al. 2021) (Tables [Link], [Link], [Link] in supporting information).

## 7 Limitations and Future Directions

Importantly, a limitation of the Hybrid‐Delphi method is that it is dependent on the experience, knowledge, and perspectives of the participants (Mead and Moseley 2000). In order to address this limitation, as well as support diversity and representation across the participant groups, we used a purposive sampling process in which we shared the study information with a wide variety of disability organisations. That said, the present sample was relatively homogeneous, with a large range of physical and sensory abilities not accounted for. The community expert group was smaller in number (*n* = 6) with three participants identifying as autistic or living with an intellectual or developmental disability, and all communicated independently. To address these limitations, future research in the dance and disability paradigm should work to equally engage dancers who live with physical disabilities as they hold a unique perspective and life experience that may contrast those who are primarily neurodivergent. Another possible limitation is in the design of the groups. Rather than combining all experts into a heterogenous group, we chose to engage each cohort of experts separately. This format aims to reduce potential challenges around group psychology (Mead and Moseley 2000) and was more conducive to the virtual focus group setting as smaller group sizes are better facilitated in an online format. Finally, future studies should include the development of a dissemination plan to share these recommendations with the dance community using a process that is co‐designed and delivered alongside dancers who identify as living with autism, intellectual, or developmental disability, dance, and rehabilitation professionals.

## 8 Conclusion

This study examined common practices in dance instruction through the combined constraints model of motor development and the social model of disability that highlighted the interactive relationship of the characteristics of (i) the dancer (physical and affective), (ii) the task and instruction, and (iii) the environment and culture of the dance setting, including the ways in which each of these factors may shape participation for dancers who identify as autistic or living with intellectual or developmental disability. The result of using this Hybrid‐Delphi method was a comprehensive list of actionable recommendations that are based on the priorities and experiences of persons with disabilities. The recommendations are applicable to a variety of education and dance settings and can be selected and explored by dance educators, schools, and organisations to guide the development of both inclusive dance and education pedagogies and spaces. Finally, each recommendation is presented as an invitation to further examine and reflect on the location of ableism in the broader culture of the dance community, specifically in dance instruction and environments.

## Ethics Statement

The ethics protocol was approved by the Research Ethics and Compliance Board (Fort Gary campus) of the University of Manitoba (HE2022‐0284).

## Consent

All participants voluntarily consented to participate in this research.

## Conflicts of Interest

The authors declare no conflicts of interest.

## Supporting information


Table S7

Table S8

Table S9

## Data Availability

The data that support the findings of this study are available on request from the corresponding author. The data are not publicly available due to privacy or ethical restrictions.

## References

[jar70060-bib-0001] ADAPT . 2023. “Associated Dance Arts for Professional Teachers.” https://www.adaptsyllabus.com/.

[jar70060-bib-0002] Albright, A. C. 2011. Choreographing Difference: The Body and Identity in Contemporary Dance. Wesleyan University Press.

[jar70060-bib-0003] Anderson, B. 2020. “Overcoming and Denial: Disability and Modern Dance in the United States.” Dance Research Journal 52, no. 3: 58–75. 10.1017/S0149767720000364.

[jar70060-bib-0004] Aujla, I. , and S. Needham‐Beck . 2020. “Subjective Well‐Being Among Young Dancers With Disabilities.” International Journal of Disability, Development and Education 67, no. 5: 563–570. 10.1080/1034912X.2019.1615607.

[jar70060-bib-0005] Aujla, I. J. , and E. Redding . 2013. “Barriers to Dance Training for Young People With Disabilities.” British Journal of Special Education 40, no. 2: 80–85. 10.1111/1467-8578.12021.

[jar70060-bib-0006] CSC . 2019. “Cecchetti Society of Canada.” https://cecchetticanada.com/.

[jar70060-bib-0007] DiPasquale, S. , and C. Kelberman . 2018. “An Integrative Dance Class to Improve Physical Function of People With Developmental and Intellectual Disabilities: A Feasibility Study.” Arts & Health 12, no. 3: 236–249. 10.1080/17533015.2018.1537295. 31038425

[jar70060-bib-0008] Fancourt, D. , and S. Finn . 2019. “What Is the Evidence on the Role of the Arts in Improving Health and Well‐Being? A Scoping Review.” Nordic Journal of Arts, Culture and Health 2, no. 1: 77–83. 10.18261/issn.2535-7913-2020-01-08. 32091683

[jar70060-bib-0009] Getchell, N. , and L. Gagen . 2006. “Adapting Activities for all Children: Considering Constraints Can Make Planning Simple and Effective.” Palaestra 22, no. 1: 20.

[jar70060-bib-0010] Hansen, N. , and C. Philo . 2007. “The Normality of Doing Things Differently: Bodies, Spaces, and Disabilty Geography.” Tijdschrift voor Economische en Sociale Geografie 98, no. 4: 493–506. 10.1111/j.1467-9663.2007.00417.x.

[jar70060-bib-0011] Hasson, F. , S. Keeney , and H. McKenna . 2000. “Research Guidelines for the Delphi Survey Technique.” Journal of Advanced Nursing 32, no. 4: 1008–1015. 10.1046/j.1365-2648.2000.t01-1-01567.x. 11095242

[jar70060-bib-0012] Humphrey‐Murto, S. , and M. de Wit . 2019. “The Delphi Method—More Research Please.” Journal of Clinical Epidemiology 106: 136–139. 10.1016/j.jclinepi.2018.10.011. 30352274

[jar70060-bib-0013] Kattai, H. R. 2023. “Reflections on Sport, Disability, and the Need for Adaptive Physical Activity to Evolve: Growing Up.” In Reflexivity and Change in Adaptive Physical Activity: Overcoming Hubris, edited by D. Goodwin and M. Connolly . Routledge. 10.4324/9781003196747.

[jar70060-bib-0014] Ladwig, J. C. , E. M. Broeckelmann , K. M. Sibley , et al. 2023. “A Synthesis of the Characteristics of Dance Interventions Engaging Adults With Neurodevelopmental Disabilities: A Scoping Review.” Disability and Rehabilitation 46, no. 10: 1–8. 10.1080/09638288.2023.2217384. 37272778

[jar70060-bib-0015] Ladwig, J. C. , E. M. Broeckelmann , K. M. Sibley , et al. 2024. “When We Dance It's Never Just Dancing…: Understanding the Experiences and Perspectives of Adult Dancers With Neurodevelopmental Disability.” European Journal of Adapted Physical Activity 17: 14. 10.5507/euj.2024.0112024.

[jar70060-bib-0016] Landeta, J. , J. Barrutia , and A. Lertxundi . 2011. “Hybrid Delphi: A Methodology to Facilitate Contribution From Experts in Professional Contexts.” Technological Forecasting and Social Change 78, no. 9: 1629–1641. 10.1016/j.techfore.2011.03.009.

[jar70060-bib-0017] Leadbitter, K. , K. L. Buckle , C. Ellis , and M. Dekker . 2021. “Autistic Self‐Advocacy and the Neurodiversity Movement: Implications for Autism Early Intervention Research and Practice.” Frontiers in Psychology 12, no. 782: 635690. 10.3389/fpsyg.2021.635690. 33912110 PMC8075160

[jar70060-bib-0018] Mastrominico, A. , T. Fuchs , E. Manders , et al. 2018. “Effects of Dance Movement Therapy on Adult Patients With Autism Spectrum Disorder: A Randomized Controlled Trial.” Behavioral Sciences 8, no. 7: 61. 10.3390/bs8070061. 29966313 PMC6071290

[jar70060-bib-0019] McCarthy‐Brown, N. 2017. Dance Pedagogy for a Diverse World: Culturally Relevant Teaching in Theory, Research and Practice. McFarland.

[jar70060-bib-0020] Mead, D. , and L. Moseley . 2000. “The Use of the Delphi as a Research Approach.” Nurse Researcher 8, no. 4: 4–23. 10.7748/nr2001.07.8.4.4.c6162.27707347

[jar70060-bib-0021] Morris, M. L. , M. Baldeon , and D. Scheuneman . 2015. “Developing and Sustaining an Inclusive Dance Program: Strategic Tools and Methods.” Journal of Dance Education 15, no. 3: 122–129. 10.1080/15290824.2015.1056301.

[jar70060-bib-0022] Newell, K. M. 1986. “Constraints on the Development of Coordination.” In Motor Development in Children: Aspects of Coordination and Control, edited by M. G. Wade and H. T. A. Whiting , 341–360. Martinus Nijhoff Publishers.

[jar70060-bib-0023] Oliver, M. 2013. “The Social Model of Disability: Thirty Years on.” Disability & Society 28, no. 7: 1024–1026. 10.1080/09687599.2013.818773.

[jar70060-bib-0024] Oliver, M. , and C. Barnes . 2010. “Disability Studies, Disabled People and the Struggle for Inclusion.” British Journal of Sociology of Education 31, no. 5: 547–560. 10.1080/01425692.2010.500088.

[jar70060-bib-0025] Oliver, M. , and C. Barnes . 2012. The new Politics of Disablement. Palgrave and MacMillan.

[jar70060-bib-0026] PHEC . 2023. “Physical Health and Educafion Canada: Dance Education.” https://phecanada.ca/activate/dance‐education.

[jar70060-bib-0027] RAD . 2023. “Royal Academy of Dance (Canada).” https://ca.royalacademyofdance.org/.

[jar70060-bib-0028] Stone, E. , and M. Priestley . 1996. “Parasites, Pawns and Partners: Disability Research and the Role of Non‐Disabled Researchers.” British Journal of Sociology 47, no. 4: 699–716. 10.2307/591081. 8969126

[jar70060-bib-0029] Suppo, J. , and T. Swank . 2019. “Steps to Inclusion—Dance Steps That Is!” Dance Education in Practice 5, no. 4: 17–23. 10.1080/23734833.2019.1672471.

[jar70060-bib-0030] Taussig, R. 2020. Sitting Pretty: The View From My Ordinary Resilient Disabled Body. HarperOne.

[jar70060-bib-0031] Teixeira‐Machado, L. , and J. M. DeSantana . 2019. “Effect of Dance on Lower‐Limb Range of Motion in Young People With Cerebral Palsy: A Blinded Randomized Controlled Clinical Trial.” Adolescent Health, Medicine and Therapeutics 10: 21–28. 10.2147/AHMT.S177867. 30988649 PMC6441460

[jar70060-bib-0032] Terada, K. , A. Satonaka , Y. Terada , and N. Suzuki . 2017. “Training Effects of Wheelchair Dance on Aerobic Fitness in Bedridden Individuals With Severe Athetospastic Cerebral Palsy Rated to GMFCS Level V.” European Journal of Physical and Rehabilitation Medicine 53, no. 5: 744–750. 10.23736/S1973-9087.17.04486-0. 28178772

[jar70060-bib-0033] Tomasic, M. 2014. “Developing Curricula and Assessemnt Tools for the Physically Intergrated Dance Class.” In 2013 VSA Intersections: Arts and Special Education Exemplary Programs and Approaches, edited by S. M. Malley , 182–202. John F. Kennedy Center for the Performing Arts.

[jar70060-bib-0034] Whatley, S. 2007. “Dance and Disability: The Dancer, the Viewer and the Presumption of Difference.” Research in Dance Education 8, no. 1: 5–25. 10.1080/14647890701272639.

[jar70060-bib-0035] Whatley, S. 2008. Moving Matters: Supporting Disabled Dance Students in Higher Education. Conventry University. https://www.invisibledifference.org.uk/media/papers/movingmatters_1.pdf.

[jar70060-bib-0036] Wright, A. , R. Roberts , G. Bowman , and A. Crettenden . 2019. “Barriers and Facilitators to Physical Activity Participation for Children With Physical Disability: Comparing and Contrasting the Views of Children, Young People, and Their Clinicians.” Disability and Rehabilitation 41, no. 13: 1499–1507. 10.1080/09638288.2018.1432702. 29382235

