# Supplementary material for: Community‐Informed Recommendations to Developing Inclusive Dance Opportunities: Engaging Community, Dance, and Rehabilitation Experts Using a Hybrid‐Delphi Method
Source: J Appl Res Intellect Disabil. 2025 May 5;38(3):e70060. doi: 10.1111/jar.70060 (PMC12051090; doi:10.1111/jar.70060)
Supplement: Supplementary file 1 — Table S7 [file JAR-38-e70060-s003.pdf]

Table 7

Supporting Recommendations: Strategies for adapting class structure and environment*

1. The Space:

- Consider providing/loaning noise cancelling headphones, ear buds or foam ear plugs.
- Access to equipment that allows teachers to adjust and/or dim lighting, adjust audio levels, or to amplify their voices (microphone) is recommended.
- Ensure that the space is large enough to facilitate personal space for all dancers and assistants.
- Define a quiet area outside the dance space for persons to go if they need to self-regulate before/during/after the class. Consider including dimmed lighting, seating such as bean bag chairs, and zones of regulation signage.

2. Class sizes:

- Be mindful of the size and dynamic of a class (considering physical, communication, sensory, and emotional regulation needs) to allow for more person-centred instruction.

3. Accessibility:

- Dancers using wheelchairs or other mobility devices/aids should be able to enter the building and/or school, studio and/or classroom, have easy access to the washrooms, and move around the class and studio freely and easily.
- Is the type of flooring compatible with their mobility aids (e.g., carpet)? Are tactile markers available for students with low vision?
- Ensure that building entrances and drop-off locations are accessible and clearly marked.
- Discourage the use of perfumes, scented cleaning supplies, or hair products in the building.

4. Consistency and flexibility of instruction:

- It is critical that the teacher understand the need for and demonstrate flexibility in their delivery of the class and communication.
- Use a consistent yet flexible lesson plan that considers the complexity of the movement and the amount of energy required throughout the class (e.g., consider your class plan as an outline rather than a strict plan).
- Communicate change and introduce any new elements in the class to both the dancers and assistants at the beginning of the class or before a transition to prepare them for the transition.
- Maintain a balance between focused activities (teacher directed movement), improvisation/creativity (movement exploration) and integrate rest breaks to hydrate and recover and/or recharge from the activity.

5. Consider sensory needs and transitions:

- Adjust the traditional expectations of the dance class (loosening of the rules) by allowing natural expressions of joy, nervousness, stress, etc. (e.g., stimming, tics), accepting movement that may not be performed exactly as taught, allowing students to sit if they cannot stand for the duration, to move/fidget during instruction, and understand that some dancers may not be quiet/settle into class.
- Reduce visual and auditory information such as fluorescent lighting, mirrors, clutter (e.g., posters, props, etc.) and volume of music can serve as a significant barrier to participation for some individuals.
- Consider sensitivities to fabrics, fit of clothing/shoes into consideration when deciding costuming and allow families/dancers the flexibility to wear to class what is most comfortable for them.

(continued on next page)

- To promote self-regulation/participation consider dancers’ sensory processing strengths, needs, and differences related to auditory, visual, tactile, and olfactory senses. For example, provide a visual for the routine they are learning, promote listening to the music for the routine before the class or before practising the choreography in class.
- Incorporate the use of visual tools such as visual schedules and/or visual timers to provide a sense of predictability, comfort, and consideration for those who require more processing time.

6. Facilitate participation:

- Be encouraging.
- Develop a class/studio culture and learning environment that is accepting and welcoming. Consider how you are creating a space, encouraging independence, and advocating for the dancers' needs within the class.
- Ensure that the dancers’ voices are heard when planning activities/choreography. Involve dancers in performance planning to ensure adequate time to process and prepare.
- Provide or enlist instructional support such as an additional teacher or class assistant to meet the needs of those participants requiring more support.
- Consider the skills and ability of who is in the environment to support your group (assistants, carers). Consider how they will be engaged in the class.
- Be mindful of the language used around emotions and regulation. Model and support dancers' learning the range of their emotions and language around how they feel.

7. Observation:

- Provide options to cover viewing windows (and mirrors) and space for family and others to wait that is outside of the dance space.

8. Communication:

- To decrease the demand for in-the-moment processing, increase predictability, comfort, and reduce anxiety for all dancers, build in communication strategies to facilitate a welcoming, inclusive environment. E.g., provide a video example of the class before the first class, email upcoming changes to dancers and assistants.

Note. *(*) Strategies as they relate to the items listed in Table 3.*
