# Supplementary material for: Community‐Informed Recommendations to Developing Inclusive Dance Opportunities: Engaging Community, Dance, and Rehabilitation Experts Using a Hybrid‐Delphi Method
Source: J Appl Res Intellect Disabil. 2025 May 5;38(3):e70060. doi: 10.1111/jar.70060 (PMC12051090; doi:10.1111/jar.70060)
Supplement: Supplementary file 2 — Table S8 [file JAR-38-e70060-s001.pdf]

Table 8

Supporting Recommendations: Strategies for Inclusive/Adaptive Instruction*

1. Adaptations for different abilities of dancers:
• For dancers who may need physical assistance use a hand under hand approach so that the dancer has control of the movement.<br>• Always ask permission before providing physical assistance.
2. Feedback and direction:
• Be encouraging.<br>• If a student is struggling with a sequence/step, acknowledge their progress. Then suggest a way to improve the step OR provide an adaptation.<br>• Praise your dancers with specificity ('I like your focus' vs. 'good job' or, for children 'good job reaching with your hand!' vs. 'good job') and use their names often.<br>• With adults, it may be more important to share the whys of the feedback and how this will help the movement.
3. Active vs. Passive Instruction:
• Model the action and energy you want to see from your dancers.<br>• Dance full out, engage your dancers, laugh together.<br>• Include verbal cues in your demonstration.
4. Flexible Instruction:
• Acknowledge dancers' disabilities. Proactively make accommodations and/or adapt the choreography to include their strengths.<br>• Integrate dancers' voices into ongoing modifications of class planning. For example, asking 'how are things going' during (when appropriate) and after the class, followed by troubleshooting if needed.<br>• Support the autonomy of the dancers by offering opportunities to choose what they would like to do (A or B) and to contribute to the class or the piece of choreography.<br>• Allow for variations on a theme of movement, as opposed to requiring all dancers to look the same.<br>• Allow the exploration of movements on their own before stepping into facilitate or cue movement.<br>• Break down (scaffold) complex movements/activities into smaller steps before putting a movement sequence together, as needed. Use repetition to ensure understanding and 'solidify' movement patterns.<br>• Be open to adapting when and where to pivot instructions in the class (i.e., if you've tried something 3+ times and it still isn't working, try a different approach), and adapt accommodations such as volume of music or lighting, and vary the use of space (e.g., circle, lines, in personal space).<br>• Provide clear proactive transition instructions/cues and expectations. Anticipate and allow for additional wait time and processing time between providing instruction and expecting a response (particularly if the dancer uses AAC; e.g., wait 30-60 sec).<br>• Be very careful if cueing for posture and ensure close monitoring of each dancer. Postural cues can be easily misinterpreted by any individual (including those without NDD) and have the potential to cause harm if not interpreted correctly.
5. Understand dancer's personal preferences around language, communication, space, etc.
• Be conscious of the language you choose regarding the dancers' bodies. Be open to modifying the words you choose. Use inclusive language such as 'travel to stage right' vs. 'walk to stage right'.<br>• Normalize alternate methods of communication. For dancers who use augmented and alternative forms of communication (AAC) teachers should have information on what AAC is and what that might look like in the dance class (e.g., student may communicate nonverbally vs verbally; they may use technology for this or a symbol board or even sign language.).

- To ensure adequate teacher preparation, begin collecting information regarding dancer's abilities, needs, and wants prior to a class starting. Ask the dancer or carer/support person what cues or strategies they have found to be helpful in other programs or classes.
- Learn dancers' individual personal space preferences (e.g., closeness to other dancers, carers/support persons, teacher) and tactile preferences (e.g., none, deep pressure vs. light touch, certain locations only if needed, etc.).
- Learn dancers' communication preferences and facilitate a variety of forms of communication in the class (e.g., AAC, ASL, visual communication tools, somatic cues). Ensure there is consistent open line of communication for individuals to update the teacher as needed.

6. Attend to dancers physical and social cues:

- Pay attention to and respond to the somatic (physical, behavioural) cues of the dancers. An teacher's response to these cues can help to boost or calm the energy of the group.
- Be mindful of signs that a dancer requires rest, change in position, hygiene break, sustenance, or medical attention.
- Integrate breaks for hydration, regulation, medication needs, and/or accommodation to leave early for pre-scheduled rides.
- Check-in with dancers before they join/re-join the class to see how they are feeling and/or how their body is feeling.
- Consider using visual aids to help dancers to express how they are feeling (e.g., a visual scale, zones of regulation).

7. Incorporate regulation skills such as breathing exercises.

8. How to assist dancers in class:

- Use straightforward language that is clear, concise, and positive. Provide instructions that explain what TO do, rather than what NOT to do.
- Ask dancers permission to use your hands or touch for correction.
- If you feel someone needs guidance traveling in the space offer your hand or arm and let them take your hand/s or your arm in a way that is comfortable for them.
- Allow dancers to understand and respond to an instruction before providing a verbal cue or assisting physically (e.g., don't provide physical or verbal cueing when a simple gesture is sufficient).
- Create a buddy system where dancers and assistants work together.

9. Pace of the class:

- For a one-hour class a warmup (section) that is 5-15 minutes long is ideal.
- Maintaining the intended flow of the class without distractions/disruptions is the highest priority.
- Engage proactive strategies to refocus dancers on the task at hand.
- Co-teachers and/or assistants can assist with individualized attention (questions and challenges) to ensure a lesson progresses as intended.

10. Understanding and self-regulation:

- If a student isn't listening to you or is having a bad day - don't take it personally. Practice self-regulation of your emotions (teacher) so that you can provide the dancer with a helpful response.
- Allow the use of fidgets in class while listening to instruction or waiting.

11. Positive and Energetic:

- Give clear and concrete instructions and celebrate successes!
- Encourage dancers to do what they can and of what they can do better.
- Ensure that the language used is clear and specific for the duration of the class.

12. Support participation and learning:

- Consider how students are configured in the space and the size of the space. Ensure clear sight lines, adequate personal space for dancers as well as carers/support persons, opportunity to follow someone, etc.
- Mirroring (facing the dancers when instructing) and moving with the dancers are helpful teaching strategies that allow for direct eye contact and encourage participation.
- Use a variety of music and change it up every so often.
- Repetition is a valuable tool for learning dance sequences. Learning is enhanced when video recordings are used and shared with the dancers for home practice and review.
- The incorporation of props can be a beneficial way for all students to learn concepts such as beat and rhythm, size, and reach, sharp and smooth. The sensory nature of props can be helpful for understanding movement and musical qualities, visualizing patterns, and engaging the focus of the dancers.

13. Be prepared for when injury or dysregulation occur:

- It is recommended that teachers have training in both basic first aid and mental health first aid.
- Trauma informed training for teachers can be helpful as it will provide a deeper understanding of behaviours and can inform when and how much to assist when a dancer is dysregulated or is in a medical crisis.

14. Be self-aware:

- Be aware of your attending behaviours and how you otherwise communicate as an teacher (e.g., tone and volume of voice, use of body language and facial expressions).

15. Cultivate a culture of acceptance:

- Presume that each dancer is competent.
- Ensure those who do not identify as having a developmental disability (in the class) are respectful and accepting of other dancers' disclosures, needs and differences.
- Facilitate general learning of diverse communication, mobility, sensory, and cognitive needs, and preferences for participants who do not identify as having a developmental disability.

16. Incorporate inclusivity into instruction.

17. Visibility and visual communication:

- It is important to ensure the teacher can be seen by all, including hard of hearing or DEAF dancers, as well as ASL interpreters.
- Using a mirrored approach (the teacher is always facing students) will help with both eye contact and lip reading.

18. Foster familiarity and understanding:

- Introduce students and assistants to one another at the start of class.
- With the permission of the dancers' share any pertinent behaviours that might cause anxiety, such as repetitive behaviours or sounds that might be misinterpreted by others as negative, with each dancer (one to one).

Note. (*) Strategies as they relate to the items listed in Table 4.
