# Supplementary material for: Community‐Informed Recommendations to Developing Inclusive Dance Opportunities: Engaging Community, Dance, and Rehabilitation Experts Using a Hybrid‐Delphi Method
Source: J Appl Res Intellect Disabil. 2025 May 5;38(3):e70060. doi: 10.1111/jar.70060 (PMC12051090; doi:10.1111/jar.70060)
Supplement: Supplementary file 3 — Table S9 [file JAR-38-e70060-s002.pdf]

Table 9

Supporting Recommendations: Information for Assistants*

1. Be an active assistant:
   - How assistants participate in the class has a direct impact on the dancer they assist, as well as the overall energy of the class. If the assistant is not engaged, the student will disengage as well, affecting class participation.
   - Understand your role as an assistant in the class. Be aware of when to provide support and when to step back to allow independent participation.
   - The role of an assistant during the class is to support the goals of the dancer, to allow dancers to participate at their own level and in their own time, and to avoid distracting the class as this can interfere with the sensory input/instructions of the teacher.
   - Assistants should be prepared to act with genuine compassion and awareness, to be able to actively "listen" to the dancers, and to respond mindfully and effectively.
   - It is important to include and recognize the assistants as participants and encourage their full participation.
   - Language matters. Clarify current language expectations (what is helpful/harmful) around disability and identity; support the use of pronouns. Understand that identity disclosure is at the discretion of the individual (dancer). For example, do they identify as an autistic vs person with ASD, etc.

2. To dance with, not for:
   - It is critical that assistants dance WITH the dancers, not FOR them. Provide instruction to assistants on a support hierarchy such as i) wait and allow independent initiation of movement, ii) gestures to indicate movement/provide physical cueing, iii) verbal prompts/cues.
   - Assistants should participate in the dance movements as much as possible themselves (modeling the movements).
   - If/when working with a dancer who uses a power wheelchair ASK if they would like assistance with driving their wheelchair BEFORE assuming control of their wheelchair.

3. Provide support and ongoing learning:
   - Include support persons and assistants in an orientation (or meet and greet) with the teacher/s and dancers before the first class.
   - Respectful and clear communication between the teacher and the assistant is valuable. Teachers must communicate clear expectations to all assistants.
   - Make sure support persons are aware of the needs of the dancer(s) as identified by them/family/support person (as appropriate), as they will relate to the dance class.
   - If/when appropriate provide education and/or demonstration on how to safely assist dancers with the movement of limbs, head positions and or body shapes of their partner within the structure of the class.
   - Regular 'check-ins' between the teacher/s and assistant/support persons are recommended. These can be an opportunity to collaboratively deepen understanding of disability and related behaviours, develop teaching and assisting strategies, and support ongoing development for both parties.
   - Open a discussion of what biases, preconceptions, assumptions they might bring to their facilitation. Emphasize that its ok to make mistakes, and we will all make mistakes. 'We' are a team - and supporting each other is also important.
   - Teachers/Schools should consider that sometimes an assistant will just not be a good fit for a dancer/the program, and that it will always be better to go with less support or to work harder to find the right support, than it is to continue with an ineffective or disrespectful support person.

4. Modeling behaviour:

- Assistants are encouraged to get to know the dancer/s they support through informal check-ins before and after the class.
- Support persons might need information on how to incorporate themselves into choreography (or if they will be choreographed in) and how to safely rehearse or review at home (if preferred/required).

5. Be Respectful:

- The role of an assistant during the class is to support the goals of the dancer, to allow dancers to participate at their own level and in their own time.
- Assistants should avoid distracting the class as this can interfere with sensory input and the instructions of the teacher.

6. Ongoing Learning:

- Consider providing a training session (video/virtual/in-person) to ensure that all assistants understand class structure, their support role, orient them to the environment (e.g., if needing to support in the washroom, if taking a break outside the dance studio, getting a drink of water, etc.) and what sensory tools/places are available.
- To encourage ongoing education for assistants (and all participants who do not identify as having a developmental disability) offer resources (handouts, video infographics, an orientation) describing common myths and misunderstandings about disability (physical and neuro-cognitive variants). Emphasize that no one group is homogenous. This may cover all types of NDD diagnoses and behaviours including emotional triggers, physical limitations, and helpful strategies.
- Consider providing information/resources for trauma-centred training for both support persons and teachers.
- Depending on the needs of the group or dancer encourage ongoing training on dancers' communication strengths and needs (e.g., use of AAC or not).

7. Communication:

- This can be very personal, some dancers have cues that they use at home or at school, if assistants are informed of what these are, then they can likely adapt much quicker and easier to the class.
- If working with hard of hearing or DEAF dancers, it is helpful to provide ASL interpreters access to song lyrics for specific choreography in advance of the class.
- Open communication between dancers and support persons is encouraged. For adults, acknowledging expertise is important. Lived experience is often downplayed in educational contexts, but the person is the expert in their disability and you, as an assistant, bring a different kind of expertise.

Note. (*) Strategies as they relate to the items listed in Table 5.
